# Accurate staging of axillary lymph nodes from breast cancer patients using a novel molecular method

**DOI:** 10.1038/bjc.2011.350

**Published:** 2011-08-30

**Authors:** T Osako, T Iwase, K Kimura, K Yamashita, R Horii, F Akiyama

**Affiliations:** 1 Division of Pathology, The Cancer Institute of Japanese Foundation for Cancer Research, Tokyo, Japan; 2 Division of Pathology, The Cancer Institute Hospital of Japanese Foundation for Cancer Research, 3-8-31, Ariake, Koto-ku, Tokyo 135-8550, Japan; 3 Division of Breast Oncology, The Cancer Institute Hospital of Japanese Foundation for Cancer Research, Tokyo, Japan

**Keywords:** breast cancer, sentinel node biopsy, non-sentinel lymph node metastasis, one-step nucleic acid amplification assay, histopathological examination; micrometastasis

## Abstract

**Background:**

The one-step nucleic acid amplification (OSNA) assay is a molecular-based lymph-node metastasis detection procedure that can assess a whole node and yields semi-quantitative results for the detection of clinically relevant nodal metastases. We aimed to determine the performance of the OSNA assay as an accurate nodal staging tool in comparison with routine histological examination.

**Methods:**

Subjects comprised 183 consecutive patients with pT1-2 breast cancer who underwent axillary dissection after positive sentinel-node (SN) biopsy with the OSNA assay. Of these, for non-SN evaluation, 119 patients underwent OSNA assay evaluation, whereas 64 had single-section histology. We compared the detection rates of non-SN metastasis and upstaging rates from the SN stage according to the American Joint Committee on Cancer staging between the OSNA and histology cohorts.

**Results:**

OSNA detected more cases of non-SN metastases than histology (OSNA 66/119, 55.5% *vs* histology 13/64, 20.3% *P*<0.001), particularly micrometastases (36/119, 30.3% *vs* 1/64, 1.6% *P*<0.001). Total upstaging rates were similar in both cohorts (20/119, 16.8% *vs* 9/64, 14.1%, *P*=0.79).

**Conclusion:**

OSNA detects a far greater proportion of non-SN micrometastases than routine histological examination. However, upstaging rates after axillary dissection were not significantly different between both cohorts. Follow-up of the OSNA cohort is required to determine its clinical relevance.

In breast cancer, the number of axillary lymph-node metastases is the most powerful prognostic factor (Fisher *et al*, 1983) and nodal tumour burden is a continuous variable of prognosis (Vinh-Hung and Storme, 2006; Weaver *et al*, 2011). Sentinel lymph-node (SN) biopsy is the standard axillary staging procedure for patients with clinically node-negative early-stage breast cancer (Lyman *et al*, 2005). Current guidelines recommend completion axillary lymph-node dissection (CALND) for patients with SN metastases (Lyman *et al*, 2005; NCCN, 2011). This procedure is considered valuable in achieving regional control and obtaining accurate nodal staging for the selection of adjuvant chemotherapy and radiation therapy, which leads to improved survival of node-positive patients (Moore and Kinne, 1997). In addition, identifying tumour spread to non-SNs beyond the SNs seems to be an independent determinant of patient outcome (Jakub *et al*, 2011).

Recently, however, the American College of Surgeons Oncology Group Z-0011 randomised trial, which was designed to compare survival in SN-positive patients who did or did not undergo CALND, has found no difference in locoregional recurrence rates and survivals between the two study arms (Giuliano *et al*, 2010, 2011). However, a more careful consideration is required for accurate node staging after CALND to determine treatments for SN-positive patients. Patients enrolled in this trial were limited, and poor prognostic patients who have three or more positive SNs were not included. Therefore, there is a potential role for avoiding CALND in a selected and limited group of SN-positive patients, but eligibility criteria and the role of adjuvant therapies need to be further elucidated (Pepels *et al*, 2011).

Conventional histopathological examinations are non-standardised and limited in their ability to detect metastases accurately because of the partial evaluation of a node. This may lead to underestimation of nodal staging. Since the late 1940s, it has been clear that the more sections of axillary lymph nodes are examined, the more metastases are identified (Saphir and Amromin, 1948). For evaluation of SNs, a step-sectioning procedure with or without immunohistochemical staining has been performed to prevent false-negative diagnoses (Giuliano *et al*, 1995). However, for evaluation of non-SNs, a more approximate procedure than that for SNs, such as single-section histology without immunohistochemical staining, has been adopted. Although the false-negative rate and underestimation of the metastasis volume can be reduced by serial sectioning and immunohistochemistry (Umekita *et al*, 2002; Reed *et al*, 2004; Tan *et al*, 2008), this procedure forces a heavy workload and cost on technicians and pathologists.

The one-step nucleic acid amplification (OSNA) assay (Sysmex Corporation) is a rapid molecular detection procedure that analyses lymph-node metastases by detection and amplification of cytokeratin 19 (CK19) mRNA (Tsujimoto *et al*, 2007). This assay can assess the whole lymph node and yields semi-quantitative results for detection of clinically relevant nodal metastases. According to the cutoff value for CK19 mRNA, OSNA assay can distinguish macrometastases and micrometastases from low expression levels corresponding to isolated tumour cells. Several clinical trials have shown that in at least half of the lymph nodes tested, the OSNA assay could accurately detect lymph-node metastases in breast cancer, at a rate comparable to that of conventional pathological examination (Visser *et al*, 2008; Schem *et al*, 2009; Tamaki *et al*, 2009; Feldman *et al*, 2011; Snook *et al*, 2011). Furthermore, we have shown that the OSNA whole-node assay detects more SN metastases, particularly micrometastases than does frozen-section histology using a 2-mm-sectioned node (Osako *et al*, 2011).

Therefore, we had hypothesised that the OSNA whole-node assay for non-SNs in addition to SNs enables the classification of accurate nodal staging for breast cancer patients. In this retrospective cohort study, we compared the performance of the OSNA assay with that of routine permanent histology for detection of non-SN metastases among patients with positive SN biopsy who have undergone a CALND.

## Patients and methods

### Patients and tumours

Subjects comprised consecutive patients with clinically and ultrasonographically node-negative pT1-2 breast cancer who had undergone CALND after a positive SN biopsy with the OSNA assay between April 2009 and September 2010 at the Cancer Institute Hospital (Tokyo, Japan). Exclusion criteria were as follows: (1) SN identification without using the radioisotope (RI) tracer, (2) previous excision of primary tumour, (3) heterochronous ipsilateral breast cancer recurrence, and (4) neoadjuvant drug therapy. In September 2009, we switched the detection of further non-SN metastases from a permanent histology method to the OSNA assay. Therefore, there are two distinct cohorts: earlier patients who were assessed by permanent histology (histology cohort) and later patients by the OSNA assay (OSNA cohort) for detection of non-SN metastases.

Pathological T and N classification was classified according the Cancer Staging Manual of the American Joint Committee on Cancer (AJCC), 7th edition, 2010 (Edge *et al*, 2010). pN1mi was defined as lymph-node spread confined to micrometastasis (>0.2 to ⩽2 mm). pN1a was defined as metastasis in 1–3 axillary node(s), including at least 1 macrometastasis (>2.0 mm). pN2a was defined as metastasis in 4–9 axillary nodes, including at least 1 macrometastasis. pN3a was defined as metastasis in ⩾10 axillary nodes, including at least 1 macrometastasis or metastasis in an infraclavicular node or nodes.

Cutoffs for oestrogen and progesterone receptor positivity were 10% positive cells, irrespective of intensity. Human epidermal growth factor receptor-2 (HER2) positivity was defined as HER2 3+ (>10% of positive cells on immunohistochemistry) or HER2 gene/centromere 17 ratio ⩾2.0 on fluorescence *in situ* hybridisation.

### SN biopsy procedure

The RI tracer used was 1.5 mCi ml^−1^ of 99mTc-phytate. One day before surgery, the tracer was injected into the intradermal and subdermal space in the area of the tumour and the retro-tumoural space. In all cases, lymphoscintigraphy was performed 1 h after the injection. In addition, 2–3 ml of vital dye, indigocarmine, was injected in the peri-tumoural space or areola at the time of surgery. Before surgery for the primary tumour, SNs were identified using a hand-held gamma-probe with guidance from the staining of vessels and nodes. All removed SNs were intraoperatively evaluated by the OSNA assay. When the SN(s) was positive, CALND was performed immediately.

### Permanent histology for non-SN examination

All non-SNs were sliced in half along the long axis after formalin fixation. One of the cut surfaces was examined after haematoxylin and eosin staining. Approximately five to seven nodes were embedded in paraffin in one cassette. Immunohistochemical staining was not used for evaluation of non-SNs.

We reviewed the non-SN specimens and classified them into three categories according to the 7th AJCC Staging Manual (Edge *et al*, 2010): positive, macrometastasis; positive, micrometastasis; negative, isolated tumour cells (⩽0.2 mm) or no tumour cell. When cancer cells were observed in multiple lymph nodes, the priority order in determining the non-SN status was macrometastasis, then micrometastasis.

### The OSNA assay for examination of SN and non-SN

The OSNA assay for lymph nodes has been described in detail in a previous publication (Tsujimoto *et al*, 2007). In brief, after removal of the extranodal tissue, whole lymph nodes were homogenised with 4 ml of lysis buffer solution (Lynorhag, Sysmex Corporation) and centrifuged at 10 000 × **g** at room temperature. In all, 2 *μ*l of the supernatant was analysed using the RD-100i system (Sysmex Corporation), an automated molecular detection system using a reverse transcription loop-mediated isothermal amplification method (Notomi *et al*, 2000), and with the LynoampBC kit (Sysmex Corporation). The degree of amplification was detected using a by-product of the reaction, pyrophosphate (Mori *et al*, 2001). The resulting change in turbidity upon precipitation of magnesium pyrophosphate was in turn correlated with CK19 mRNA copy number per *μ*l of the original lysate by a standard curve, which was established beforehand with three calibrators containing different CK19 mRNA copy numbers. A standard positive control sample containing 5000 copies per *μ*l of CK19 mRNA and a negative control sample not containing any CK19 mRNA were used for quality assurance in every assay run. Lymph nodes that exceeded the specified maximum weight of 600 mg were cut into two or more pieces and processed as separate nodes. Up to four lymph nodes can be analysed in one run.

The number of CK19 mRNA copies per *μ*l was calculated, and based on this number, the result (positive/negative) was assessed in accordance with the cutoff level determined by Tsujimoto *et al* (2007). The OSNA assay can classify a positive node into three categories: (++), (+), and (+I; positive with reaction inhibited); criteria are shown in Table 1. In this study, OSNA (++) and (+I) were considered to be equivalent to AJCC macrometastasis, and OSNA (+) to AJCC micrometastasis. Moreover, when multiple pieces or lymph nodes were positive, the priority order for determining the SN and non-SN status was (++), (+I), and (+).

All SNs and a small number of non-SNs surrounding SNs were assessed intraoperatively. Almost all non-SNs in CALND specimens were assessed postoperatively. The non-SNs were put in tubes, and were immediately frozen at −80 °C in the deep freezer (My Bio Cube, Nihon Freezer Co., Ltd, Tokyo, Japan). The frozen non-SNs were assessed in the same manner as fresh nodes at a later date.

### Statistical analyses

First, to compare the characteristics of SN-positive patients between the histology and OSNA cohorts, we performed Student's *t*-test for the age, Mann–Whitney test for the number of SNs or non-SNs removed, and *χ*^2^-tests for the other characteristics. Second, to compare detection rates of non-SN metastases, including macrometastases and micrometastases, between the histology and OSNA cohorts, we performed the two-population *z*-test for non-SN metastases (positive *vs* negative), macrometastases (histological macrometastasis *vs* OSNA (++) or (+I)), and micrometastases (histological micrometastasis *vs* OSNA (+)). Third, to compare the impact on overall AJCC staging after the non-SN assessment with permanent histology *vs* that with the OSNA assay, we performed the two-population *z*-test for upstaging rates from the SN stage of both cohorts. A *P*-value of <0.05 was considered significant and confidence intervals (CIs) were set at the 95% level. Computation of these analyses was performed using the statistical software R (version 2.10.1, http://www.r-project.org/).

## Results

### Patient characteristics

Between April 2009 and September 2010, 792 consecutive patients underwent SN biopsy with the OSNA whole-node assay and did not fulfil the exclusion criteria. Of these, 184 (23.2%) were diagnosed as SN positive, and 183 underwent CALND consequently. Subjects of this study comprised 64 patients in the histology cohort and 119 patients in the OSNA cohort. The demographic characteristics of both cohorts are presented in Table 2. All patients were Japanese women and the two cohorts were well balanced for all patient characteristics.

### Detection of non-SN metastasis

Non-SNs were found to be positive for metastasis more frequently in the OSNA cohort than in the histology cohort (histology 13/64, 20.3%, 95% CI; 11.7–32.6% *vs* OSNA 66/119, 55.5%, 95% CI; 46.1–64.5% *P*<0.001) (Table 3). We found no significant difference in the frequency of histological macrometastasis and OSNA (++) or (+I) in non-SNs (12/64, 18.8%, 95% CI; 10.5–30.8% *vs* 30/119, 25.2%, 95% CI; 17.9–34.2% *P*=0.420). However, we found significant difference in the frequency of histological micrometastasis and OSNA (+) in non-SNs (1/64, 1.6%, 95% CI; 0.1–9.5% *vs* 36/119, 30.3%, 95% CI; 22.3–39.5% *P*<0.001). Median numbers of positive non-SNs were 2 (interquartile range, 1 and 12) in the histology cohort and 1 (interquartile range, 1 and 2) in the OSNA cohort.

### Stage migration after non-SN examination

In the histology cohort, 9.5% (2/21) of patients with pN1mi(sn) and 17.1% (7/41) of those with pN1a(sn) were upstaged after non-SN examinations (Table 4). In the OSNA assay cohort, 14.0% (7/50) of patients with pN1mi(sn), 16.9% (11/65) of those with pN1a(sn), and 50.0% (2/4) of those with pN2a(sn) were upstaged after non-SN examination (Table 5). Total upstaging rates were similar in the histology and the OSNA cohorts (histology 9/64, 14.1%, 95% CI; 7.0–25.5% *vs* OSNA 20/119, 16.8%, 95% CI; 10.8–25.0% *P*=0.79).

## Discussion

As far as we know, this is the first report in which all entire SNs and non-SNs were evaluated by a molecular assay without using any histopathological examination. In terms of correlation between the results of molecular assay and histopathological metastatic size, we defined OSNA (++) and (+I) as equivalent to AJCC macrometastasis, and OSNA (+) as equivalent to AJCC micrometastasis in this study. According to a three-level CK19 immunohistochemistry-based histopathology method, the cutoff value of 5000 copies per *μ*l in the OSNA assay is 85% concordant with macrometastasis. On the basis of a serial-sectioning experiment, the cutoff value of 250 copies per *μ*l in the OSNA assay can detect metastatic foci of 0.3–0.4 mm^3^ (Tsujimoto *et al*, 2007). Therefore, the OSNA assay can distinguish macrometastases and micrometastases from low-volume metastases corresponding to AJCC-isolated tumour cells. However, there is a lack of evidence regarding OSNA (+I) defined as a macrometastasis in this study. The nucleic acid amplification method is occasionally inhibited by unknown material, but this inhibition is mitigated in the diluted samples. We considered OSNA (+I) as indicative of high-volume metastasis corresponding to AJCC macrometastasis, because CK19 mRNA was ⩾250 copies per *μ*l in the 10-fold more diluted samples.

This study demonstrated that the OSNA whole-node assay detected significantly more cases of non-SN metastases than routine histopathological examination. The OSNA assay detected significantly higher number of cases with micrometastases which appeared to be missed by routine histology. This is reasonable considering that a single-section histology analyses only a limited part of the lymph node, whereas the OSNA assay can thoroughly evaluate total metastatic volume in a lymph node.

The non-SN-positive rate of the present histology cohort (20%) was lower than that in other reports (30–50%) (Rahusen *et al*, 2001; Travagli *et al*, 2003; Viale *et al*, 2005). The number of observed sections of non-SN has some range from a single section to the detailed examinations in these reports. This variation in detection sensitivity for non-SN metastases is one of the reasons that node staging accuracy has not been standardised yet.

The OSNA assay does not show superiority to the routine histological examination for node staging if the AJCC guidelines are applied. The upstaging rates from the SN stage based on the current AJCC staging system were similar in the histology and OSNA cohorts in this study. This is because the OSNA assay detected one or two positive non-SN(s) containing micrometastasis.

At the present time, it is unclear whether identifying more non-SN micrometastases with the OSNA assay has an impact on the clinical outcome of SN-positive patients. Follow-up of the OSNA cohort is required to determine its clinical relevance. However, a recent study has shown that identifying tumour spread to non-SNs beyond SNs seems to be an important determinant of patient outcome independent of the number of involved nodes (Jakub *et al*, 2011). In addition, a prospective study with long-term follow-up has shown that occult metastases detected by the molecular analysis have prognostic impact (Verbanac *et al*, 2010). Thus, there is a possibility that patients who were identified non-SN micrometastases with the OSNA assay have a worse survival rate than do those who were not identified.

The current AJCC nodal staging is based on the results of histopathological examinations. However, histopathological examinations may not be a sufficient ‘gold standard’ for nodal staging (Snook *et al*, 2011), because they are non-standardised and usually limited in their ability to detect metastases accurately due to the partial observation of a node. On the other hand, the OSNA assay can evaluate a whole lymph node in a standardised manner. Thus, with long-term data on the outcome of patients who underwent the OSNA assay, this assay could be the ‘gold standard’ for nodal staging. In this study, SN biopsy with RI tracer rigorously separated axillary nodes into SNs and non-SNs, and evaluation by the OSNA whole-node assay provided semi-quantitative results of each whole SN and non-SN. Therefore, follow-up of the OSNA cohort can clarify more accurately the prognostic impact of SN, non-SN, and total axillary metastatic volume. This could lead to a new nodal staging system using OSNA results.

In conclusion, the OSNA whole-node assay detects a far greater proportion of non-SN metastases than do single-section permanent histology in patients with a positive SN biopsy. However, in terms of the AJCC staging system, upstaging rates from the SN stage were similar in the histology and OSNA cohorts. Follow-up of the OSNA cohort is required to clarify the prognostic implications of this technique; this may lead to the establishment of a new breast cancer staging and contribute to personalised cancer care.

## Figures and Tables

**Table 1:** Definition of positive lymph nodes by the one-step nucleic acid amplification assay

		**CK19 mRNA (copy per *μ*l) in diluted sample**	
		**<250**	**⩾250**
CK19 mRNA (copy per *μ*l) in measurement sample	⩾5000	Positive (++)	
	250–5000	Positive (+)	
	<250	Negative	Positive (+I)

Abbreviations: CK19=cytokeratin 19; (+I)=positive with reaction inhibited.

**Table 2:** Patient characteristics of the permanent histology and one-step nucleic acid amplification (OSNA) assay cohorts

	**Histology**		**OSNA**		
**Characteristics**	**No.**	**%**	**No.**	**%**	***P-*value**
No. of patients	64	100	119	100	
					
*Age*					0.13
Median (range)	56 (39–81)		53 (27–86)		
					
*Menstrual status*					0.079
Premenopausal	21	32.8	55	46.2	
Postmenopausal	43	67.2	64	53.8	
					
*Breast surgery*					0.25
Conservative	32	50.0	70	58.8	
Mastectomy	32	50.0	49	41.2	
					
*SN identification*					0.84
Radioisotope alone	10	15.6	20	16.8	
Radioisotope plus dye	54	84.4	99	83.2	
					
*No. of SNs removed*					0.92
Median (range)	2 (1–8)		2 (1–10)		
Interquartile range	1, 3		1, 3		
					
*No. of positive SNs*					0.61
1	53	82.8	91	76.5	
2	8	12.5	20	16.8	
⩾3	3	4.7	8	6.7	
					
*SN status*					0.28
(+)	21	32.8	50	42.0	
(+I)	15	23.4	18	15.1	
(++)	28	43.8	51	42.9	
					
*AJCC SN stage*					0.46
pN1mi(sn)	21	32.8	50	42.0	
pN1a(sn)	41	64.1	65	54.6	
pN2a(sn)	2	3.1	4	3.4	
					
*Axillary surgery*					0.13
Level I	10	15.6	30	25.2	
Level II or III	54	84.4	89	74.8	
					
*No. of non-SNs removed*					0.11
Median (range)	17 (8–40)		15 (6–33)		
Interquartile range	13, 21		13, 19		
					
*Pathological T classification*					0.57
pT1mi	2	3.1	4	3.4	
pT1a	2	3.1	10	8.4	
pT1b	8	12.5	20	16.8	
pT1c	33	51.6	56	47.1	
pT2	19	29.7	29	24.4	
					
*Histological type*					0.59
Ductal	57	89.1	110	92.4	
Lobular	2	3.1	4	3.4	
Special types	5	7.8	5	4.2	
					
*Nuclear grade*					0.22
1	19	29.7	54	45.4	
2	27	42.2	39	32.8	
3	11	17.2	17	14.3	
Lobular/special type	7	10.9	9	7.6	
					
*Lymphovascular invasion*					0.23
−	29	45.3	65	54.6	
+	35	54.7	54	45.4	
					
*Fat infiltration*					0.86
−	8	12.5	16	13.4	
+	56	87.5	103	86.6	
					
*Oestrogen receptor status*					0.43
−	12	18.8	17	14.3	
+	52	81.3	102	85.7	
					
*Progesterone receptor status*					0.97
−	24	37.5	45	37.8	
+	40	62.5	74	62.2	
					
*HER2 status*					0.53
−	55	85.9	106	89.1	
+	9	14.1	13	10.9	

Abbreviations: AJCC=American Joint Committee on Cancer; HER2=human epidermal growth factor receptor-2; (+I)=positive with reaction inhibited; SN=sentinel lymph node.

**Table 3:** Detection of non-sentinel lymph-node metastasis in the permanent histology and one-step nucleic acid amplification (OSNA) assay cohorts

	**Histology**			**OSNA**				
	**No.**	**%**	**95% CI**		**No.**	**%**	**95% CI**	***P*-value**
Positive	13	20.3	11.7–32.6%	Positive	66	55.5	46.1–64.5%	<0.001
Macrometastasis	12	18.8	10.5–30.8%	(++) or (+I)	30	25.2	17.9–34.2%	0.42
Micrometastasis	1	1.6	0.1–9.5%	(+)	36	30.3	22.3–39.5%	<0.001
								
Negative	51	79.7		Negative	53	44.5		

Abbreviations: CI=confidence interval; (+I)=positive with reaction inhibited.

**Table 4:** Upstaging rates in the permanent histology cohort according to the 7th American Joint Committee on Cancer Staging System

		**Overall axillary stage**								
		**pN1mi**		**pN1a**		**pN2a**		**pN3a**		
**Sentinel lymph- node stage**	**No.**	**No.**	**%**	**No.**	**%**	**No.**	**%**	**No.**	**%**	**Upstaging rate (%)**
pN1mi (sn)	21	19	90.5	1	4.8	1	4.8	0	0	9.5
pN1a (sn)	41	—	—	34	82.9	4	9.8	3	7.3	17.1
pN2a (sn)	2	—	—	—	—	2	100	0	0	0
All	64	19	29.7	35	54.7	7	10.9	3	4.7	14.1

**Table 5:** Upstaging rates in the one-step nucleic acid amplification assay cohort according to the 7th American Joint Committee on Cancer Staging System

		**Overall axillary stage**								
		**pN1mi**		**pN1a**		**pN2a**		**pN3a**		
**Sentinel lymph- node stage**	**No.**	**No.**	**%**	**No.**	**%**	**No.**	**%**	**No.**	**%**	**Upstaging rate (%)**
pN1mi(sn)	50	43	86.0	6	12.0	0	0	1	2.0	14.0
pN1a(sn)	65	—	—	54	83.1	9	13.8	2	3.1	16.9
pN2a(sn)	4	—	—	—	—	2	50.0	2	50.0	50.0
All	119	43	36.1	60	50.4	11	9.2	5	4.2	16.8
